# Establishing standardized transthoracic echocardiography reference ranges for mouse models: insights into the impact of anesthesia, sex, and age

**DOI:** 10.3389/fcvm.2025.1695034

**Published:** 2025-12-19

**Authors:** Manuela A. Oestereicher, Christopher S. Ward, Elida Schneltzer, Susan Marschall, Helmut Fuchs, Valerie Gailus-Durner, Ghina Bou About, Mohammed Selloum, Hamid Meziane, Michelle Stewart, Lydia Teboul, Clare Norris, Dale Pimm, Marina Kan, Federico López Gómez, Robert Wilson, Mayra Monroy, Sheraz Pasha, Eva Zabrodska, Jan Prochazka, David Pajuelo Reguera, Zuzana Nichtova, Yann Herault, Sara Wells, Helen Parkinson, Jason D. Heaney, Radislav Sedlacek, Xiang Gao, Martin Hrabe de Angelis, Nadine Spielmann

**Affiliations:** 1 Institute of Experimental Genetics and German Mouse Clinic, Helmholtz Center Munich, German Research Center for Environmental Health, Neuherberg, Germany; 2 Department of Integrative Physiology, Baylor College of Medicine, Houston, TX, United States; 3 Université de Strasbourg, CNRS, INSERM, Institute de la Clinique de la Souris, CELPHEDIA, PHENOMIN, Illkirch, France; 4 The Mary Lyon Centre and MRC Harwell, Harwell Campus, Oxfordshire, United Kingdom; 5 European Molecular Biology Laboratory-European Bioinformatics Institute, Wellcome Trust Genome Campus, Hinxton, Cambridgeshire, United Kingdom; 6 Advanced Technology Cores, Baylor College of Medicine, Houston, TX, United States; 7 Czech Centre for Phenogenomics, Institute of Molecular Genetics of the Czech Academy of Sciences, Prague, Czech Republic; 8 Department of Molecular and Human Genetics, Baylor College of Medicine, Houston, TX, United States; 9 SKL of Pharmaceutical Biotechnology and Model Animal Research Center, Collaborative Innovation Center for Genetics and Development, Nanjing Biomedical Research Institute, Nanjing University, Nanjing, China; 10 Chair of Experimental Genetics, School of Life Science Weihenstephan, Technische Universität München, Freising, Germany; 11 German Center for Diabetes Research (DZD), Neuherberg, Germany

**Keywords:** transthoracic echocardiography (TTE), mouse cardiac reference ranges, anesthesia effects on cardiac function, C57BL/6N mouse model, cardiovascular phenotyping

## Abstract

**Introduction:**

Mouse models play a critical role in cardiology research, offering valuable insights into the molecular mechanisms, genetics, and potential treatments for cardiovascular diseases. However, the ability to transfer findings in mice between studies is limited by the absence of standardized protocols and valid reference values for the assessment of normal cardiac function in mice. This study aims to establish comprehensive transthoracic echocardiography (TTE) reference ranges for mice, particularly focusing on C57BL/6N wild-type controls.

**Methods:**

The study, which includes data from over 15,000 mice through the International Mouse Phenotyping Consortium (IMPC), highlights how variables such as sex, age, body weight, and anesthesia impact TTE parameters.

**Results:**

The findings showed that anesthesia is the primary predictor of variability in cardiac function. Isoflurane- and tribromoethanol-anesthetized mice presented with modified cardiac function compared with conscious mice. In addition, we observed minimal sex differences in cardiac morphology and function, except for small variations influenced by anesthesia. The effects of aging on cardiac function were modest, characterized by a decrease in heart rate and subtle changes in ventricular dimensions without evidence of pathological remodeling, possibly attributable to disease-free cardiovascular aging.

**Discussion:**

Validation of the reference ranges across multiple mouse strains showed that these values provide a reliable baseline for experiments involving cardiac function in mice. The data underscore the importance of using anesthesia-specific reference values when interpreting TTE results, ensuring robust comparisons in genetic and pharmacological studies. These reference ranges serve as quality assurance tools for future cardiac studies in mice, offering insights into typical TTE parameter values, supporting the detection of experimental perturbations, and contributing to more effective translation of findings from mice to humans.

## Introduction

Transthoracic echocardiography (TTE) is a vital non-invasive tool for diagnosing, monitoring, and guiding treatment in a wide range of heart conditions. TTE reference ranges from large healthy populations have become an increasingly common and valuable tool for diagnostic decision-making in clinical medicine (1–5).

Mouse models play a critical role in cardiology research, offering valuable insights into the molecular mechanisms, genetics, and potential treatments for cardiovascular diseases (6–8). Their widespread use in cardiology stems from the ease of genetic manipulation in mice, their relatively short generation times and lifespans, and the similarity of many physiological and pathological processes between mice and humans (9).

Advancements in echocardiographic equipment and transducers have enabled their successful adaptation from humans to rodents (10, 11). Hence, the establishment of TTE reference ranges of healthy mice creates a benchmark for normal cardiac physiology and will improve the understanding of normal values and variation across sex, age, or strains.

TTE reference ranges are not only fundamental to evaluate disease models by revealing how diseases or interventions impact the model, but they also benchmark animal welfare by signaling health issues when values deviate—which can aid in assessment of drug safety, revealing potential toxic or adverse effects from normal values.

TTE reference ranges allow comparison across studies by standardizing data, ensuring consistency and reproducibility of research findings. In mouse models, they have the potential to improve data interpretation, animal welfare, and research reliability toward translatability (12, 13). However, reference ranges are not very common in mice, often highly specific in their nature for the mouse model and typically based on very small numbers of mice (14–16).

Building on the recent European Society of Cardiology (ESC) position paper outlining requirements for murine echocardiography (9), we expanded these foundations using data from the International Mouse Phenotyping Consortium (IMPC). From more than 15,000 conscious and anesthetized C57BL/6N wild-type mice, we derived standardized TTE reference ranges stratified by sex and age. This large-scale, quantitative analysis establishes a generalizable framework for defining normal cardiac morphology and function in mice and provides the first validated, multicenter resource for reproducible echocardiographic phenotyping in preclinical cardiovascular research.

## Materials/methods

### The international mouse phenotyping consortium

The IMPC represents a multi-institutional and collaborative research initiative encompassing 24 major research organizations and funding agencies, distributed globally (17). The IMPC seeks to generate and phenotype a knockout mouse line for every protein-coding gene in the orthologous mouse genome (https://www.mousephenotype.org) (18). Phenotyping is carried out under the uniform operating procedures detailed in IMPReSS (International Mouse Phenotyping Resource of Standardized Screens; https://www.mousephenotype.org/impress/index), which were developed and validated during the pilot programs EUMORPHIA and EUMODIC (19).

### IMPC centers contributing transthoracic echocardiography data

IMPC data release (DR) 21 was used herein (https://ftp.ebi.ac.uk/pub/databases/impc/all-data-releases/release-21.0/). The following subset of six IMPC data–contributing centers provided TTE data in DR 21 (ethical approval details are included in parenthesis after each contributing center):

1. Baylor College of Medicine (BCM) (Institutional Animal Care and Use Committee approved license AN-5896).
2. German Mouse Clinic, Helmholtz Zentrum München (GMC) (#144-10, 15-168)
3. Medical Research Council (MRC)—Harwell (HAR) (Animal Welfare and Ethical Review Body approved licenses 70/8015 and 30/3384).
4. Institute Clinique de la Souris, Mouse Clinical Institute (ICS) (#4789-2016040511578546v2).
5. Czech Centre for Phenogenomics (CCP) (AV CR 62/2016, Academy of Sci., Czech Rep.).
6. MARC Nanjing University (#NRCMM9).

TTE data were collected from mice at one of two possible time points. For the Early Adult (EA) Pipeline, data were collected at a mean of 12 weeks with the minimum of 8 and maximum of 16 weeks of age. For the Late Adult (LA) Pipeline, data were collected at a mean of 63 weeks with the minimum of 51 and maximum of 78 weeks of age. Animal welfare was assessed routinely for all mice involved.

### Animals

This study includes data collected from inbred wild-type control animals tested as part of the IMPC data. These mice, both males and females, were on a C57BL/6N genetic background of substrains: C57BL/6NCrl (CCP, HMGU, and ICS); C57BL/6NJ (BCM) and C57BL/6NTac (HMGU, ICS, and HAR).

Non-IMPC mice were taken from three different studies: (1) The founder strain animals were taken from a study titled “The Collaborative Cross: A Recombinant Inbred Mouse Population for the Systems Genetic Era” (20) with A/J, C57BL/6J, 129S1/SvlmJ, NOD/ShiLtJ, NZO/HlLtJ, CAST/EiJ, PWK/Ph, and WSB/EiJ inbred strains (https://phenome.jax.org/projects/GMC13); (2) The Jaxwest1 project is a multisystem analysis of physiology on seven inbred strains of mice: 129S1/SvImJ, A/J, BALB/cJ, C57BL/6J, DBA/2J, NOD/ShiLtJ, and SJL/J (https://phenome.jax.org/projects/Jaxwest1); (3) Eumorphia6, a project conducted under the Eumorphia/Europhenome initiative, is a collaborative effort involving multiple European research centers focused on phenotyping and genetic research on the inbred strains 129S2/SvPas, BALB/cByJ, C3HeB/FeJ, and C57BL/6J (https://phenome.jax.org/projects/Eumorphia6).

### TTE recording

The IMPC standard operating procedure provides an overview of the conscious and anesthetized TTE procedures used by contributing centers (https://www.mousephenotype.org/impress/ProcedureInfo?procID=109).

In brief, bodyweights were taken shortly before transthoracic echocardiography. For anesthetized TTE recordings, the animal was placed in an induction chamber and anesthetized with 1.5%–3% isoflurane or injected with tribromoethanol as an injectable anesthetic. While sedated, either as part of the TTE session or as a separate preparatory procedure, the animal undergoes hair removal of the chest. With the hair removed, the animal was placed on the imaging platform with its paws taped to ECG surface electrodes and a rectal probe inserted to monitor body temperature, which was maintained at 36–37 °C. During imaging, anesthesia was adjusted to maintain proper heart rate (HR) and keep the animal from waking up.

For awake TTE examinations, the animal was firmly held by the nape (in the supine position) in the palm of one hand with the tail held tightly between the last two fingers.

To facilitate ultrasound imaging, a prewarmed ultrasound gel was placed on the chest at the area of imaging and transthoracic echocardiography recordings captured. For short-axis mode, papillary muscles were used as an anatomic point of reference. TTE measurements were performed identically across all IMPC centers following a standardized protocol. The parasternal long-axis (PLAX) view served as the starting point for high-throughput TTE diagnostics in mice, with the transducer positioned vertically and the notch oriented toward the animal's head. The probe was then rotated approximately 35° counterclockwise to visualize the aortic root and apex, ensuring clear imaging of the left ventricular (LV) anterior and posterior walls. From this position, B-mode images of the LV in PLAX were recorded. Subsequently, the parasternal short-axis (PSAX) view was obtained by rotating the transducer approximately 90° clockwise to achieve a cross-sectional view of the LV, using the papillary muscles as an anatomic landmark. Both B-mode and M-mode images were acquired, with at least three M-mode recordings per view (21).

For M-mode imaging, the acquisition axis was placed centrally through the LV at the level of the papillary muscles, following system guidelines. After imaging, the animals were removed from the platform and allowed to recover on a heating pad. All TTE measurements were analyzed offline using VisualSonics analytical software, Vevolab (VisualSonics Inc.). Quantitative parameters included left ventricular internal diameter in systole (LVIDs) and diastole (LVIDd), as well as anterior and posterior wall thicknesses in systole and diastole (LVAWs, LVPWs, LVAWd, and LVPWd). In mice, we refer to the parameters as LVIDd and LVIDs, which correspond to the clinical measurements left ventricular end-diastolic diameter (LVEDD) and left ventricular end-systolic diameter (LVESD) in patients, respectively. Papillary muscles were excluded from the traced LV boundaries (21). Derived parameters were calculated from two-dimensional M-mode images using the Teichholz formula (22): fractional shortening (FS) was calculated as FS % = [(LVIDd − LVIDs)/LVIDd] × 100. Ejection fraction (EF) was calculated as EF% = 100 × [(LVvol;d − LVvol;s)/LVvol;d] with LVvol = [7.0/(2.4 + LVID) × LVID^3^]. The stroke volume (SV) is the volume of blood pumped from one ventricle of the heart with each beat. The stroke volume of the left ventricle was obtained by subtracting end-diastolic volume (LVvol;d) from end-systolic volume (LVvol;s). Heart rate was determined from the cardiac cycles recorded on the M-mode tracing, using at least three consecutive end-systolic intervals.

Importantly, each center records significant metadata parameters according to the FAIR principles (Findable, Accessible, Interoperable, Reusable; https://www.go-fair.org/fair-principles/) including equipment manufacturer, equipment model, recording environment, anesthetic agent, and anesthetic dose. Detailed experimental protocols on the IMPC phenotyping procedures are available for general access at https://www.mousephenotyping.org/IMPReSS. Data were curated and subject to quality control at the IMPC prior to DR 21 (13th June 2024); we excluded four EA and one LA mice from the analysis because their body temperatures were outside of a normal physiological range (<36, >40 °C), two EA animals with improbable LVIDd < LVIDs values, and two additional EA mice because of biologically implausible ejection fraction levels (<25%) in the conscious state.

### Statistical methods

Bespoke methods were developed to assess TTE reference ranges and are independent of the methodologies implemented on the IMPC portal.

Data and statistical analysis was conducted using R (version 4.2.2, R Core Team 2022 (23), with figures and tables produced in *ggplot2* and *ggpubr*. Variability of all the data was assessed by using the metric coefficient of variation (COV). Visual methods (histograms), as well as a formal statistical test (Shapiro–Wilk test), were conducted to test whether the scores of the individual parameters were normally distributed. Data were separated by age, sex, and anesthesia regime, and histograms for each parameter were plotted. Reference ranges were calculated based on median, 2.5th percentile, and 97.5th percentile. In addition, the mean, standard deviation, and parameter sample size were provided to reflect the distribution of each parameter. Based on this, the 95% confidence intervals can be calculated by using mean ± 1.96 × standard deviation for each parameter.

### Relative importance of predictors

To investigate the relative importance of different regressors on the outcome variable in linear models, we applied the R package *relaimpo* developed by Groemping (24) and calculated the relative importance (based on the metric “lmg”) of four predictors, namely, anesthesia (conscious, isoflurane, and tribromoethanol), sex (males and females), body weight (BW), and age (EA and LA) in all 15,765 mice. Adjusted R^2^ depicted the total proportion of variance explained by the model with all four predictors, and the relative proportion of contribution for each predictor was shown by relative (%) of adjusted R^2^.

### Investigation of anesthesia, sex, and age effects

To investigate the effect of anesthesia on the different parameters in EA mice, we calculated a one-way analysis of variance (ANOVA) with planned comparisons of “Conscious vs. Isoflurane” and “Conscious vs. Tribromoethanol,” separated by sex, whereas “Isoflurane vs. Tribromoethanol” was not tested. These planned comparisons were used to compare conscious vs. unconscious states. The null hypothesis tested whether the two datasets originate from distributions with the same mean. *P*-values and *F*-values with degrees of freedom were calculated.

The effects of sex (female vs. male) and age (EA vs. LA) were compared using identical statistical analyses. In each case, a simple two-tailed t-test was performed and the Cohen's d effect size calculated from the *effsize* package (R library). Because of the central limit theorem (CLT) (25), the large sample sizes allowed parametric statistical testing of these effects. The biological relevance of large samples may be overstated, which is why we also calculated the effect sizes to be able to estimate this factor.

### Data availability

Current data, including those used in this study, are available to the public for download at the IMPC: https://www.mousephenotype.org/data/release; DR21: https://previous-releases-reports.s3.eu-west-2.amazonaws.com/release-21.pdf, https://ftp.ebi.ac.uk/pub/databases/impc/all-data-releases/release-21.0/.

## Results

TTE data collected by IMPC contributing centers (DR, 21) were available from 15,765 wild-type control mice, stratified as presented in Table 1 and summarized below. All the mice belonged to a C57BL/6N inbred substrain. TTE was performed on conscious mice or mice anesthetized with either isoflurane or tribromoethanol. The majority of mice (89.3% or 14,083) were tested at a mean age of 12 weeks (designated as “early adult” or EA), while the remaining 10.7% (1,682) of mice were tested at a mean age of 63 weeks (designated as LA). Sex was evenly distributed at both EA and LA time points. Raw data can be downloaded using the following link: https://ftp.ebi.ac.uk/pub/databases/impc/all-data-releases/release-21.0/. The total number of reported parameters varied slightly between mice.

**Table 1:** TTE data were available from a total of 15,765 mice, stratified by sex, age at testing (EA = 12 weeks of age; LA = mean of 63 weeks of age), and conscious state (conscious, anesthetized using isoflurane, or anesthetized using tribromoethanol).

Group	Conscious	Isoflurane	Tribromoethanol	Sum
EA female	2,699	4,633	227	7,559
EA male	2,709	3,592	223	6,524
LA female	378	496	0	874
LA male	376	432	0	808
Sum	6,162	9,153	450	15,765

### Variability assessment

A panel of 12 output parameters were collected from TTE, namely cardiac output (CO), EF, LVIDd, LVIDs, FS, HR, LVAWd, LVAWs, LVPWd, LVPWs, respiration rate (RR) and SV (parameter definition in Supplementary Table S1). In addition, the BW of each mouse was weighed before the TTE measurement, and the body temperature of anesthetized mice was measured with a rectal probe to monitor physiological functions (Supplementary Figure S1 with histograms, mean ± SD, median and 95% reference ranges, separated by sex). LVAWd and LVAWs were inconsistently collected in the IMPC and not further processed in this analysis.

**Figure 1:**
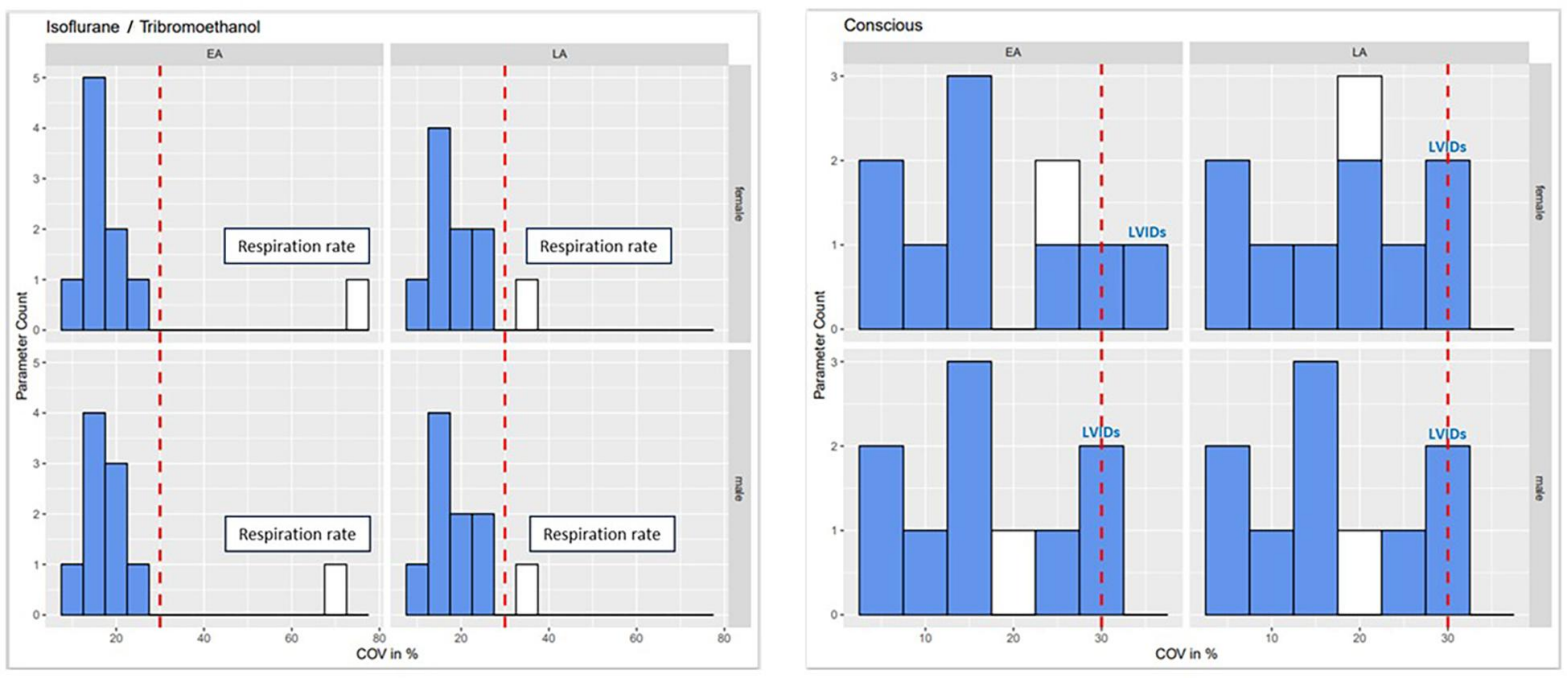
COV analysis of data, stratified by sex (female and male), anesthetic regime, and age (EA and LA), identified one parameter, respiration rate, with excess variability (COV > 30%) in LA males and females under anesthesia that was excluded from further analysis (white bar). Parameters in blue were below the COV threshold of 30% and were retained for further analysis.

In multicenter, large-scale, high-throughput programs such as the IMPC, variability in the measured values is to be expected. However, the extent of this variability dictates the sensitivity and robustness of each parameter. Variability testing was performed on all DR 21 TTE data obtained from the IMPC, independently of anesthetic agent in this analysis. For each sex, individual TTE parameters were tested for variability in EA and LA populations. The COV (100 × standard deviation/mean) assumes a parametric distribution and normalizes the variability to the most typical score (mean) but is sensitive to outliers (26). Based on this analysis, exclusion criteria were defined as any parameter with ≥30 for COV, based on Eurachem guidelines (27). Figure 1 shows that the retained parameters are all clustered closely together; however, the excluded parameter shows a wide range of variability.

Specifically, one TTE parameter, RR, exceeded the variability criteria in both sexes (male and female) and LA age but not in EA and was excluded from further analysis (Figure 1). The variability threshold was partially exceeded for LVIDs in females of EA age; however, in females of LA and males of both ages (EA and LA), the threshold was not exceeded and LVIDs was retained, whereas LVAWd and LVAWs values are exclusively shown in the Supplementary Materials.

The remaining 9 TTE parameters (CO, EF, LVIDd, LVIDs, FS, HR, LVPWd, LVPWs, and SV) consistently presented with low variability across the whole IMPC dataset, thereby providing high confidence to establish robust, generalizable reference ranges for EA and LA populations on the C57BL/6N inbred genetic background.

### Assessment of data distribution

**Figure 2:**
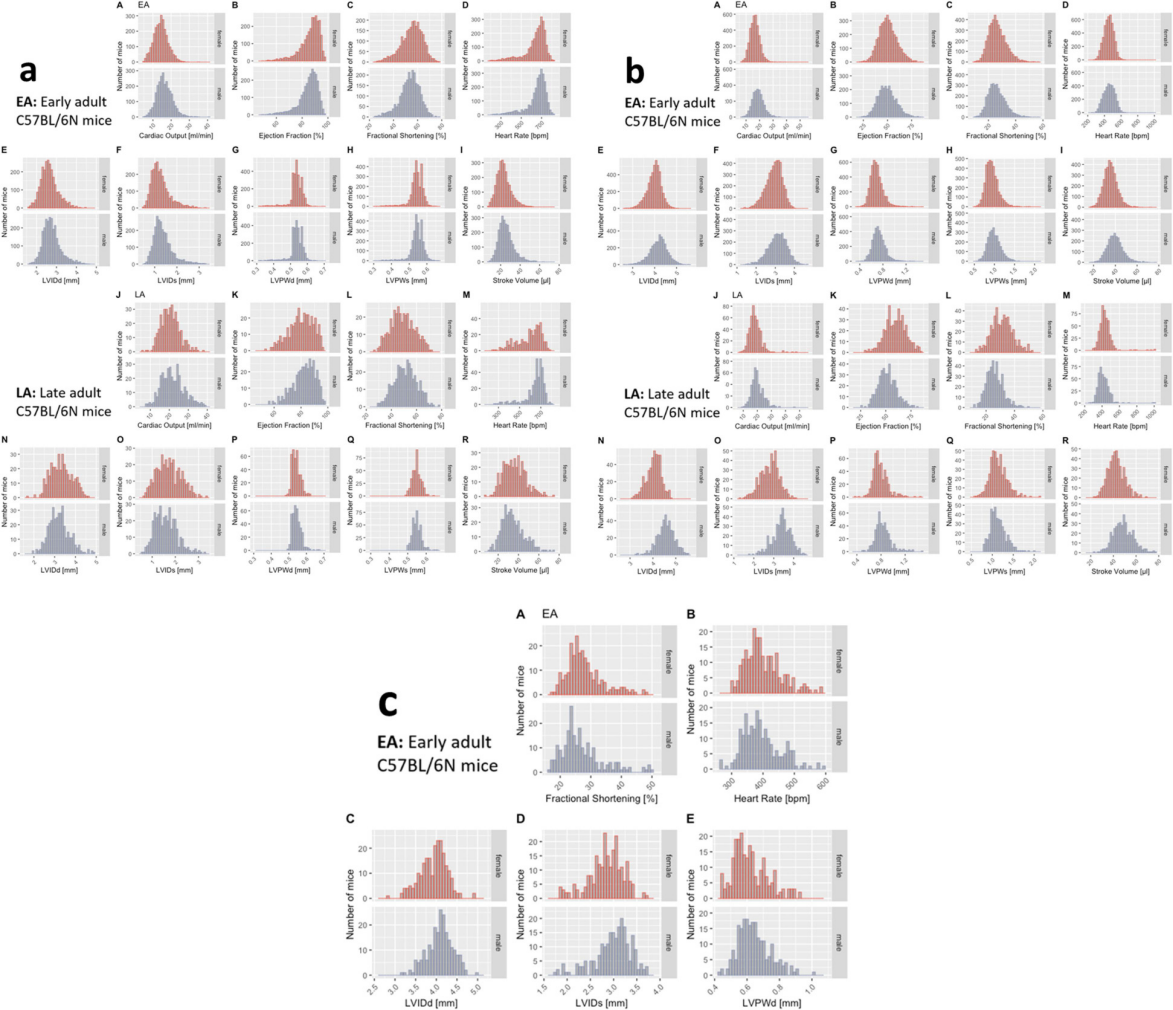
Histograms presenting the distribution of each selected TTE parameter for male (blue) and female (red) mice separately. On visual inspection, no sexual dimorphism is apparent. (**a**) Recorded in the conscious state in EA [subpanels (A–I)], and LA mice [subpanels (J–R)]. (**b**) Recorded under isoflurane anesthesia in EA [subpanels (A–I)], and LA mice [subpanels (J–R)]. (**c**) Recorded under tribromoethanol anesthesia in EA [subpanels (A–E)], with five (FS, HR, LVIDd, LVIDs, and LVPWd) out of nine TTE parameters. No late adult data are available for tribromoethanol anesthesia.

The distribution of data was assessed via histograms for the nine selected TTE parameters stratified by sex, age, and anesthetic regime (Figure 2). Under tribromoethanol anesthesia, data points were captured in EA from only five (FS, HR, LVIDd, LVIDs, and LVPWd) of the nine TTE parameters.

This visual representation of the frequency of occurrence per value in the data was useful for revealing conformity to and deviations from a normal distribution for each parameter. A visual inspection of the histograms showed that the data appeared practically normal in EA for the parameters CO, EF, LVIDd, LVIDs, FS, LVPWd, LVPWs, and SV and modestly skewed for HR in conscious mice but not under isoflurane anesthesia. Under tribromoethanol anesthesia, FS, HR, LVIDd, LVIDs, and LVPWd appeared practically normally distributed, whereas HR was modestly skewed. To assess normality mathematically, we applied the Shapiro–Wilk test, which revealed a statistically significant deviation from a normal distribution for some, but not all, TTE parameters. Table 2 presents data as median and 95% reference range (2.5th percentile and 97.5th percentile) to account for the lack of normal distribution of some parameters and to provide a consistent level of data presentation. The data are stratified by sex, age (EA and LA), and conscious state. For the sake of completeness, mean, standard deviation, and sample size are provided for the seven selected TTE parameters stratified by sex, age, and anesthetic regime in Supplementary Table S2 and LVAWd and LVAWs values in Supplementary Table S3.

**Table 2:** Median and 95% reference ranges of CO, EF, FS, HR, LVIDd, LVIDs, LVPWd, LVPWs, and SV.

Parameter	Conscious—EA	Conscious—LA	Isoflurane—EA	Isoflurane—LA	Tribromoethanol—EA	Tribromoethanol—LA
(a) Females						
Cardiac output (mL/min)	14.3 (7.0–22.5)	20.3 (12.3–31.7)	16.4 (10.1–23.8)	17.9 (12.1–27.1)	NA	NA
Ejection fraction (%)	88 (65.0–96.0)	81.1 (60.6–94.7)	49.9 (33.9–70.3)	59.9 (38.6–81.2)	NA	NA
Fractional shortening (%)	56.1 (34.9–70.1)	48.8 (32.2–67.3)	25.8 (16.2–40.7)	31.2 (17.5–48.6)	26.7 (19.6–43.1)	NA
Heart rate (bpm)	668.2 (336.8–769.2)	620.2 (343.0–751.3)	454 (342.0–556.3)	427 (339.4–549.0)	390 (315.2–538.7)	NA
LVIDd (mm)	2.59 (1.90–3.64)	3.31 (2.47–4.30)	4.01 (3.29–4.55)	4.07 (3.36–4.66)	3.97 (3.24–4.49)	NA
LVIDs (mm)	1.18 (0.68–2.36)	1.69 (0.83–2.67)	2.98 (2.04–3.65)	2.86 (1.84–3.66)	2.87 (1.9–3.4)	NA
LVPWd (mm)	0.54 (0.42–0.59)	0.54 (0.50–0.60)	0.68 (0.52–0.93)	0.77 (0.61–1.11)	0.6 (0.45–0.87)	NA
LVPWs (mm)	0.54 (0.44–0.60)	0.55 (0.50–0.59)	0.92 (0.68–1.29)	1.12 (0.78–1.56)	NA	NA
Stroke volume (µL)	22.3 (11.0–39.7)	35.7 (18.3–59.6)	35.3 (24.3–48.8)	41.9 (29.8–58.9)	NA	NA
Parameter	Conscious—EA	Conscious—LA	Isoflurane—EA	Isoflurane—LA	Tribromoethanol—EA	Tribromoethanol—LA
(b) Males						
Cardiac output (mL/min)	15.8 (8.7–25.4)	21.2 (11.5–35.3)	18.7 (11.4–27.7)	19.3 (13.0–30.4)	NA	NA
Ejection fraction (%)	86.4 (63.4–94.9)	83.4 (64.0–94.8)	48.9 (33.0–69.6)	50.9 (35.6–70.8)	NA	NA
Fractional shortening (%)	54.1 (34.1–67.9)	51.1 (34.7–67.4)	25.4 (15.8–40.2)	25.5 (16.2–39.4)	25.3 (17.4–48.4)	NA
Heart rate (bpm)	678.4 (371–774)	670.7 (360.2–753.1)	458.7 (336.0–574.5)	405.4 (326.8–520.1)	385 (302.6–528.3)	NA
LVIDd (mm)	2.7 (2.05–3.87)	3.18 (2.37–4.24)	4.21 (3.34–4.94)	4.57 (3.75–5.30)	4.11 (3.49–4.66)	NA
LVIDs (mm)	1.29 (0.76–2.54)	1.56 (0.82–2.74)	3.14 (2.13–3.99)	3.42 (2.39–4.28)	3.05 (1.91–3.63)	NA
LVPWd (mm)	0.54 (0.43–0.59)	0.54 (0.47–0.56)	0.72 (0.54–0.96)	0.81 (0.62–1.18)	0.63 (0.5–0.87)	NA
LVPWs (mm)	0.55 (0.44–0.60)	0.55 (0.50–0.61)	0.98 (0.72–1.35)	1.11 (0.83–1.56)	NA	NA
Stroke volume (µL)	24.4 (13.4–43.9)	33 (17.2–58.5)	39.3 (27.0–55.6)	48.3 (33.0–68.2)	NA	NA

Data are stratified by sex, age (EA and LA), and conscious state. Panel a: conscious, isoflurane-, and tribromoethanol-anesthetized female mice. Panel b: conscious, isoflurane-, and tribromoethanol-anesthetized male mice. Note that there were no data for LA mice anesthetized using tribromoethanol.

### Relative importance of predictors

If there are several predictors, the question naturally arises as to which predictor is more important or useful for predicting the outcome variable. For correlated predictors, the standardized coefficients may not indicate which predictor is more important. Here, the calculation of the “relative importance” of the predictors (24) is applied for the predictors of sex (males and females), anesthesia (conscious, isoflurane, and tribromoethanol), age (EA and LA), and body weight across the grand total of 15,765 mice. Anesthesia shows the highest proportion of contribution with a range of 32% (SV) to 99% (EF and FS) relative % of adjusted R^2^ for all nine TTE parameters, whereas sex, weight, and age cover rather low proportions of variance (Table 3).

**Table 3:** Adjusted R^2^ represents the total explained variance of the relaimpo model with the proportion of contribution for each predictor shown by relative % of adjusted R^2^ for anesthesia (conscious, isoflurane, or tribromoethanol anesthesia), sex (female and male), body weight, and age (EA and LA) across the total of 15,765 mice.

Parameter	Adj. R² explained model variance (%)	Anesthesia (%)	Sex (%)	Weight (%)	Age (%)
Cardiac output	18	32	16	39	13
Ejection fraction	81	99	0.3	0.3	0.3
Fractional shortening	80	99	0.3	0.3	0.3
Heart rate	62	96	1	1	2
LVIDd	80	95	0	3	2
LVIDs	82	98	0	1	1
LVPWd	52	89	1	5	5
LVPWs	75	94	0	2	4
Stroke volume	56	78	2	12	8

Anesthesia contributes with a range of 32%–99% to the adjusted R^2^ of all nine TTE parameters.

### Effect of sex

Interestingly, male and female data showed similar distributions on visual inspection (Figure 2). To test the hypothesis that there is no difference between each sex, a simple two-tailed t-test was performed independently for each anesthetic regime and age group, and Cohen's d was calculated as an effect size measure (Supplementary Figures S2–S4a and b, stratified by age). In the EA population, for some parameters, *p*-values reached a significance of <0.001; for others, we found no evidence of a difference (Supplementary Figures S2-4, Panel a). However, for all parameters, the corresponding Cohen's d value revealed small to negligible effect sizes. We, therefore, considered the possibility that the large group sizes could be overstating the biological differences between the sexes for some parameters. In the LA population, *p*-values reached a significance of <0.001 for most of the parameters, and for others, we found no evidence of a difference (Supplementary Figures S2-4, Panel b). In contrast, the corresponding Cohen's d value revealed small to negligible effect sizes for the majority of parameters; however, large effect sizes were observed in EF, LVIDd, LVIDs, and FS in mice under isoflurane and medium for SV. Here, with a relatively smaller group size than EA population, the biological differences between the sexes in the LA population were both significant and of medium or large effect sizes for some, mainly functional (and less morphological), TTE parameters. Overall, there was a difference between sexes.

### Effect of anesthetic agents

**Figure 3:**
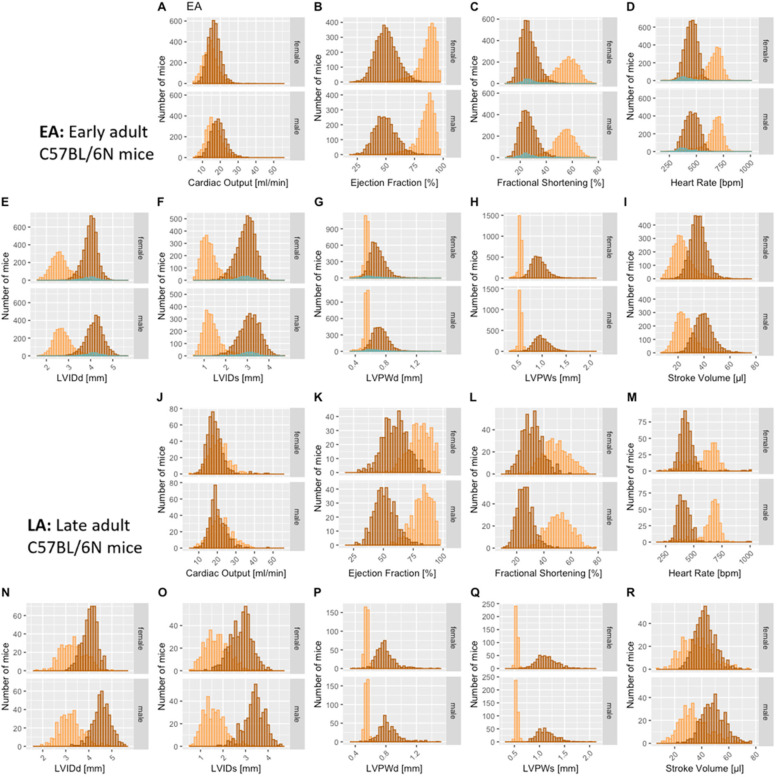
Comparison of the anesthetic regimes with the conscious state recordings. Distribution of the nine selected TTE parameters presented by histograms, stratified for female and male mice in EA [(**A**–**I**)], and LA populations [(**J–R**)]. No late adult data are available, and only FS, HR, LVIDd, LVIDs, and LVPWd parameters in EA are available for tribromoethanol anesthesia. Color code: 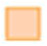 conscious, 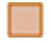 isoflurane, and 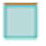 tribromoethanol anesthesia.

**Figure 4:**
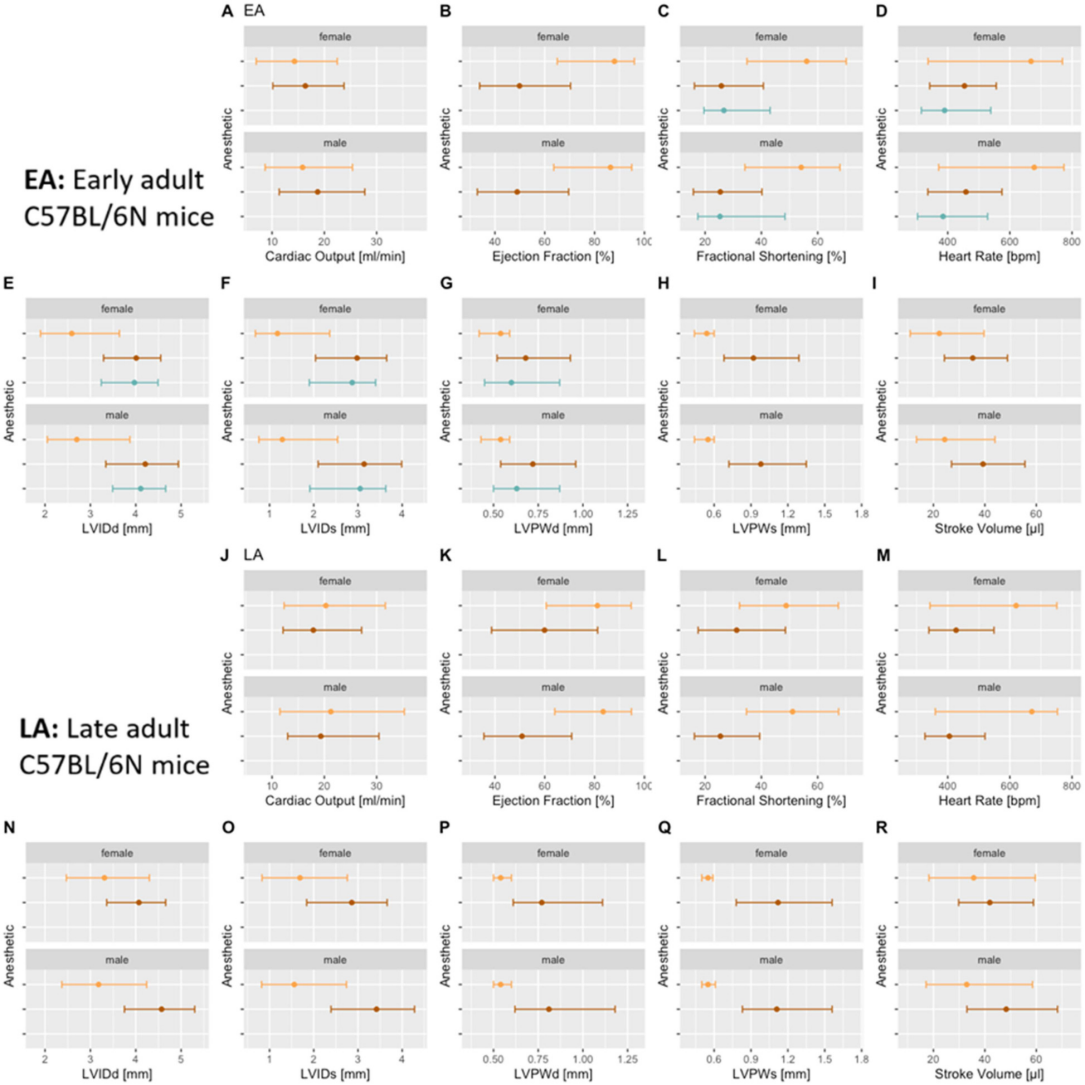
Reference ranges split by an anesthetic regimen showing median and 95% reference ranges (2.5th percentile and 97.5th percentile). Female data are directly above male data for EA [(**A**–**I**)] and LA populations [(**J**–**R**)]. No late adult data are available, and only FS, HR, LVIDd, LVIDs, and LVPWd parameters in EA are available for tribromoethanol anesthesia. Color code: 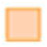 conscious, 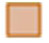 isoflurane, and 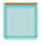 tribromoethanol anesthesia.

To investigate the effect of different anesthetic agents on cardiac conduction function and TTE profiles, conscious data stratified by sex and age are displayed for comparison with those of isoflurane or tribromoethanol data (Figure 3). Female data are placed directly above male data for ease of visualization. Figure 4 shows distinct distribution clusters for conscious, isoflurane, and tribromoethanol groups split by EA (Figures 4A–I) and LA (Figures 4J–R). As before, no data were available for tribromoethanol anesthesia in LA mice.

As expected, the physiological benchmark of the highest heart rate in conscious mice compared with anesthetized animals was observed in EA and LA populations (Figures 3D,M). To assess the differences between EA anesthetic states, we tested conscious vs. isoflurane and conscious vs. tribromoethanol groups by a one-way ANOVA with planned comparisons and observed highly significant differences between those groups, except for LVPWd in EA females conscious vs. tribromoethanol (Table 2). These data clearly show differences in TTE parameters that can be attributed to the anesthetic regime; therefore, it is essential to establish reference ranges separately by condition (conscious or anesthetized) and by anesthetic (isoflurane or tribromoethanol).

### Effect of age

Two different age groups, i.e., mean of a 12-week (minimum 8 and maximum 16 weeks)-old EA and mean of a 63-week (minimum 51 and maximum 78 weeks)-old LA, have made it possible to explore the effect of age on TTE parameters in conscious and isoflurane-anesthetized mice, stratified by sex. A two-tailed t-test was applied to test the difference between the means of EA and LA results in conscious (Table 4, Panel a) and isoflurane-anesthetized mice (Table 4, Panel b). *p*-values <0.001 were reached for all parameters, except for HR and LVPWs in conscious males, indicating high statistical significance, and the corresponding Cohen's d effect size revealed medium-to-large standardized effect sizes in CO, LVIDd, LVIDs, and SV and also in EF and FS in conscious females (Table 5, Panel a). In isoflurane-anesthetized mice, *p*-values <0.001 were reached for all parameters, except for EF in males, indicating high statistical significance, and the corresponding Cohen's d effect size revealed medium-to-large standardized effect sizes in LVIDd, LVIDs, LVPWs, and SV and also in CO, EF, FS, and LVPWd in females (Table 5, Panel b). These high-significance values with considerable effect sizes suggested that the influence of age strongly influenced the results independent of the anesthetic regime.

**Table 4:** Significant differences between the statistical comparison of conscious vs. isoflurane (*p* < 0.001) and conscious vs. tribromoethanol (*p* < 0.001 to *p* = 0.004) in female (Panel a) and male (Panel b) mice for the parameters of CO, EF, FS, HR, LVIDd, LVIDs, LVPWd, LVPWs, and SV.

Parameter	Conscious vs. isoflurane	Conscious vs. tribromoethanol
(a) Females		
Cardiac output (mL/min)	*F*(16,432) = 527.2, *p* < 0.001	No data available
Ejection fraction (%)	*F*(16,593) = 26,877.2, *p* < 0.001	No data available
Fractional shortening (%)	*F*(17,538) = 25,534, *p* < 0.001	*F*(17,538) = 683, *p* < 0.001
Heart rate (bpm)	*F*(17,533) = 9,561.5, *p* < 0.001	*F*(17,533) = 749.8, *p* < 0.001
LVIDd (mm)	*F*(17,536) = 23,387.1, *p* < 0.001	*F*(17,536) = 648.2, *p* < 0.001
LVIDs (mm)	*F*(17,537) = 27,345.1, *p* < 0.001	*F*(17,537) = 690, *p* < 0.001
LVPWd (mm)	*F*(17,537) = 4,984.7, *p* < 0.001	*F*(17,537) = 0.7, *p* = 0.42
LVPWs (mm)	*F*(16,592) = 16,533.3, *p* < 0.001	No data available
Stroke volume (µL)	*F*(16,454) = 5,677.1, *p* < 0.001	No data available
(b) Males		
CardiacoOutput (mL/min)	*F*(15,368) = 512.6, *p* < 0.001	No data available
Ejection Fraction (%)	*F*(15,533) = 23,496.7, *p* < 0.001	No data available
Fractional shortening (%)	*F*(16,504) = 22,155.6, *p* < 0.001	*F*(16,504) = 668.7, *p* < 0.001
Heart rate (bpm)	*F*(16,502) = 9,073, *p* < 0.001	*F*(16,502) = 873.4, *p* < 0.001
LVIDd (mm)	*F*(16,502) = 18,163.3, *p* < 0.001	*F*(16,502) = 501, *p* < 0.001
LVIDs (mm)	*F*(16,505) = 21,830.1, *p* < 0.001	*F*(16,505) = 574, *p* < 0.001
LVPWd (mm)	*F*(16,504) = 7,485.5, *p* < 0.001	*F*(16,504) = 5.1, *p* = 0.024
LVPWs (mm)	*F*(15,534) = 19,865, *p* < 0.001	No data available
Stroke volume (µL)	*F*(15,381) = 4,s961.7, *p* < 0.001	No data available

Test: *p*-value and F-value of one-way ANOVA with planned comparison.

**Table 5:** Significant sex-dependent differences were observed across CO, EF, FS, HR, LVIDd, LVIDs, LVPWd, LVPWs, and SV parameters in conscious (Panel a) and isoflurane-anesthetized mice (Panel b).

Parameter	*p*-value	Cohen's d	*p*-value	Cohen's d
(a)	Conscious—Males		Conscious—Females	
Cardiac output (mL/min)	*p* < 0.001	−1.24 (large)	*p* < 0.001	−1.55 (large)
Ejection fraction (%)	*p* < 0.001	0.31 (small)	*p* < 0.001	0.77 (medium)
Fractional shortening (%)	*p* < 0.001	0.26 (small)	*p* < 0.001	0.7 (medium)
Heart rate (bpm)	*p* = 0.242	0.06 (negligible)	*p* < 0.001	0.43 (small)
LVIDd (mm)	*p* < 0.001	−1.01 (large)	*p* < 0.001	−1.55 (large)
LVIDs (mm)	*p* < 0.001	−0.53 (medium)	*p* < 0.001	−1.1 (large)
LVPWd (mm)	*p* < 0.001	−0.18 (negligible)	*p* < 0.001	−0.14 (negligible)
LVPWs (mm)	*p* = 0.02	−0.09 (negligible)	*p* < 0.001	−0.15 (negligible)
Stroke volume (µL)	*p* < 0.001	−1.12 (large)	*p* < 0.001	−1.7 (large)
(b)	Isoflurane—Males		Isoflurane—Females	
Cardiac output (mL/min)	*p* < 0.001	−0.32 (small)	*p* < 0.001	−0.57 (medium)
Ejection fraction (%)	*p* < 0.001	−0.2 (small)	*p* < 0.001	−0.98 (large)
Fractional shortening (%)	*p* = 0.832	−0.01 (negligible)	*p* < 0.001	−0.75 (medium)
Heart rate (bpm)	*p* < 0.001	0.65 (medium)	*p* < 0.001	0.27 (small)
LVIDd (mm)	*p* < 0.001	−0.98 (large)	*p* < 0.001	−0.19 (negligible)
LVIDs (mm)	*p* < 0.001	−0.59 (medium)	*p* < 0.001	0.32 (small)
LVPWd (mm)	*p* < 0.001	−0.95 (large)	*p* < 0.001	−0.88 (large)
LVPWs (mm)	*p* < 0.001	−0.91 (large)	*p* < 0.001	−1.27 (large)
Stroke volume (µL)	*p* < 0.001	−1.2 (large)	*p* < 0.001	−1.08 (large)

Most comparisons yielded *p* < 0.001. Cohen's d values indicated predominantly medium-to-large effects, with the strongest sex effects under conscious conditions for CO, LVIDd, LVIDs, and SV and additionally for EF and FS in females. Under isoflurane, medium-to-large effect sizes were also present for LVIDd, LVIDs, LVPWd, LVPWs, and SV and additionally for CO, EF, and FS in females.

**Figure 5:**
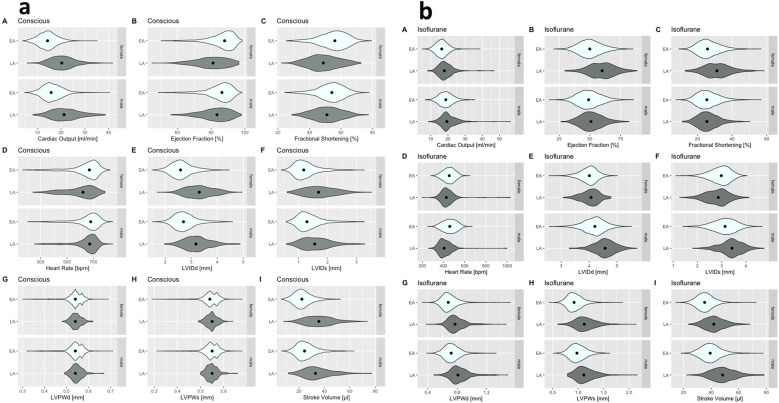
Violin plot representing full distribution of the numeric data, including the density of each TTE variable. Female data are directly above the male data for LA and EA populations for easy comparison. (**a**) Recorded in the conscious state in EA and LA. **(b**) Recorded under isoflurane anesthesia. No late adult data are available for tribromoethanol anesthesia.

In summary, Figure 5 is a graphical violin representation of the median and the full distribution of the numeric data, including the density of each TTE variable broken down by an anesthetic regimen, with the female data placed directly above equivalent male data for easy visual interpretation; corresponding numeric values are presented in Table 2. This graphical representation clearly shows that age has a strong influence with respect to the distribution and the reference ranges of nine TTE parameters.

### Effect of body weight

**Figure 6:**
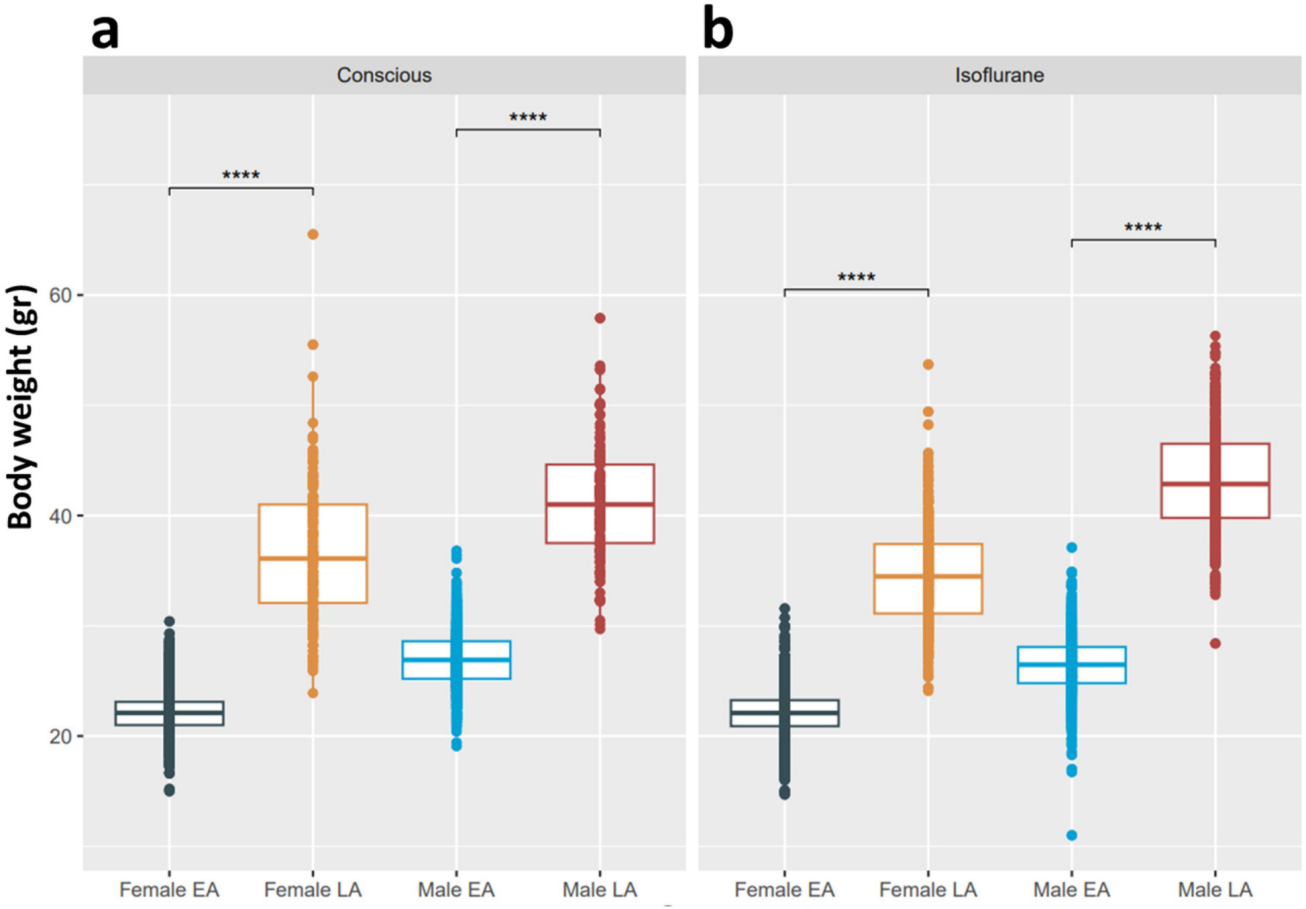
A two-tailed t-test is applied to test the difference between the means of EA and LA BW, stratified for female and male mice in (EA and LA populations split by an anesthetic regimen. (**a**) Recorded in the conscious state in EA and LA. (**b**) Recorded under isoflurane anesthesia. No BW data are available for tribromoethanol anesthesia. *p*-values < 0.001 (***) are reached for all comparisons, indicating high statistical significance between age groups.

In tandem with age, BW can also have an influence on the heart and prompted the question of how old, yet healthy C57BL/6N mice change and modify TTE profiles. Two different age groups, i.e., mean of 12-week (minimum 8 and maximum 16 weeks)-old EA and mean of 63-week (minimum 51 and maximum 78 weeks)-old LA, have made it possible to explore BW in conscious and isoflurane-anesthetized mice, stratified by sex. A two-tailed t-test was applied to test the difference between the means of EA and LA results in conscious (Figure 6a) and isoflurane-anesthetized mice (Figure 6b). *p*-values <0.001 were reached for BW in EA vs. LA, indicating high statistical significance between these two age groups independent of sex and anesthesia.

To explore the linear relationship of BW and each TTE parameter, we calculated the Pearson correlation coefficient [Pearson's R (28)], stratified by sex, age, and anesthesia. The strength of the linear relationship between TTE variables and BW was defined on the basis of user guidelines (29, 30): 0.00–0.10 negligible, 0.10–0.39 weak, 0.40–0.69 moderate, 0.70–0.89 strong, and 0.90–1.00 very strong correlations. Female data were placed directly above male data for ease of visualization.

**Figure 7:**
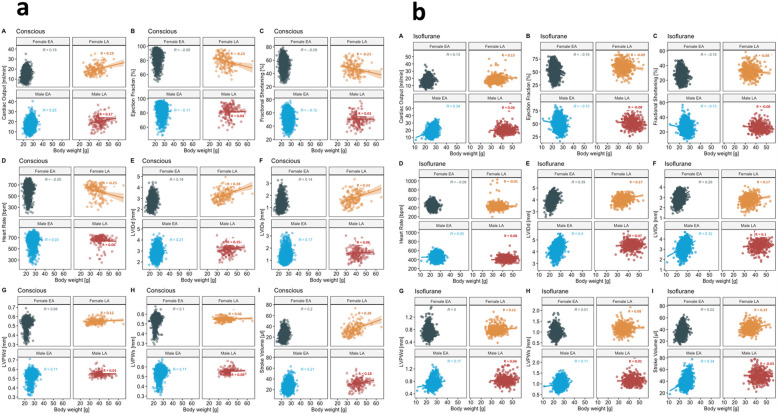
Pearson correlation with a linear regression line presenting negligible to weak correlations for all nine TTE parameters with BW (g), stratified for female and male mice in EA and LA populations split by an anesthetic regimen. (**a**) Recorded in the conscious state in EA and LA. (**b**) Recorded under isoflurane anesthesia. No late adult data are available for tribromoethanol anesthesia. LVIDd and LVIDs, left ventricular internal diameter in diastole and systole; LVPWd and LVPWs, posterior wall thicknesses in diastole and systole.

Figure 7 shows distinct distribution clusters with a regression line for conscious and isoflurane groups stratified by sex and split by EA and LA. No BW data were available for tribromoethanol anesthesia in EA and LA mice. A negligible to weak standardized Pearson correlation was reached for the entire set of TTE parameters with BW (g) in conscious and isoflurane-anesthetized mice ranging from 0.03 to 0.21 in the EA population and from 0.01 to 0.38 in the LA population (Figure 7, Panel a and b), indicating a minor role of BW; therefore, it is not entirely necessary to consider BW herein as major effect contributor.

### Validation of reference ranges using non-IMPC data

Mice characterized by the IMPC are all substrains of one commonly used inbred genetic background, C57BL/6N. To test the validity of the reference ranges reported herein beyond C57BL/6N inbred mice, we used data from representative additional inbred mouse strains from publicly available TTE data including six founder strains from a collaborative cross study (20); the Jaxwest1 project (https://phenome.jax.org/projects/Jaxwest1) with seven inbred strains of mice; and four inbred strains of the Eumorphia/Europhenome project (https://phenome.jax.org/projects/Eumorphia6). An additional dataset was included using inbred, wild-type control animals from non-IMPC studies conducted at the GMC where data are available upon request. Furthermore, we used published TTE reference ranges from the ESC position paper focusing on the appropriate echocardiographic acquisition and analysis of left ventricular function in healthy adult mice (9).

**Figure 8:**
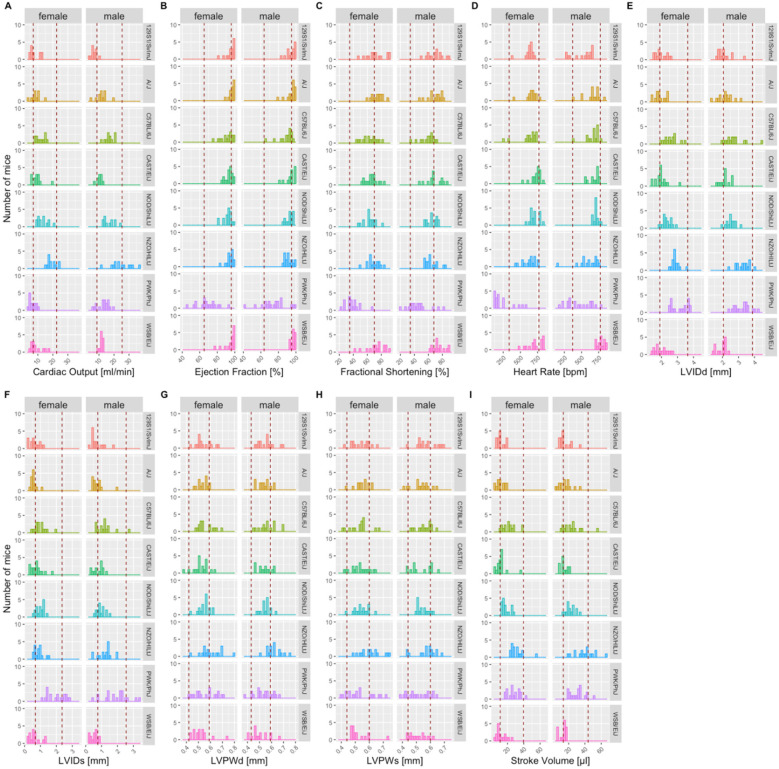
Independent, non-IMPC study on six of the founder strain mice reported in “the Collaborative Cross: A Recombinant Inbred Mouse Population for the Systems Genetic Era” (20) study, including 129S1/SvlmJ, A/J, C57BL/6J, NOD/ShiLtJ, NZO/HlLtJ, and PWK/PhJ inbred strains show a close alignment with the reference ranges reported herein for CO, EF, LVIDd, LVIDs, FS, HR, LVPWd, LVPWs, and SV based on multiple C57BL/6N substrains indicating good utility for those reference ranges. Mice were conscious, split by sex, and ∼12 weeks of age, equivalent to the IMPC EA time point. The red dotted lines depict the boundaries of the sex-specific reference ranges calculated from the C57BL/6N IMPC animals described earlier, for each parameter. LVIDd and LVIDs, left ventricular internal diameter in diastole and systole; LVPWd and LVPWs, posterior wall thicknesses in diastole and systole.

**Figure 9:**
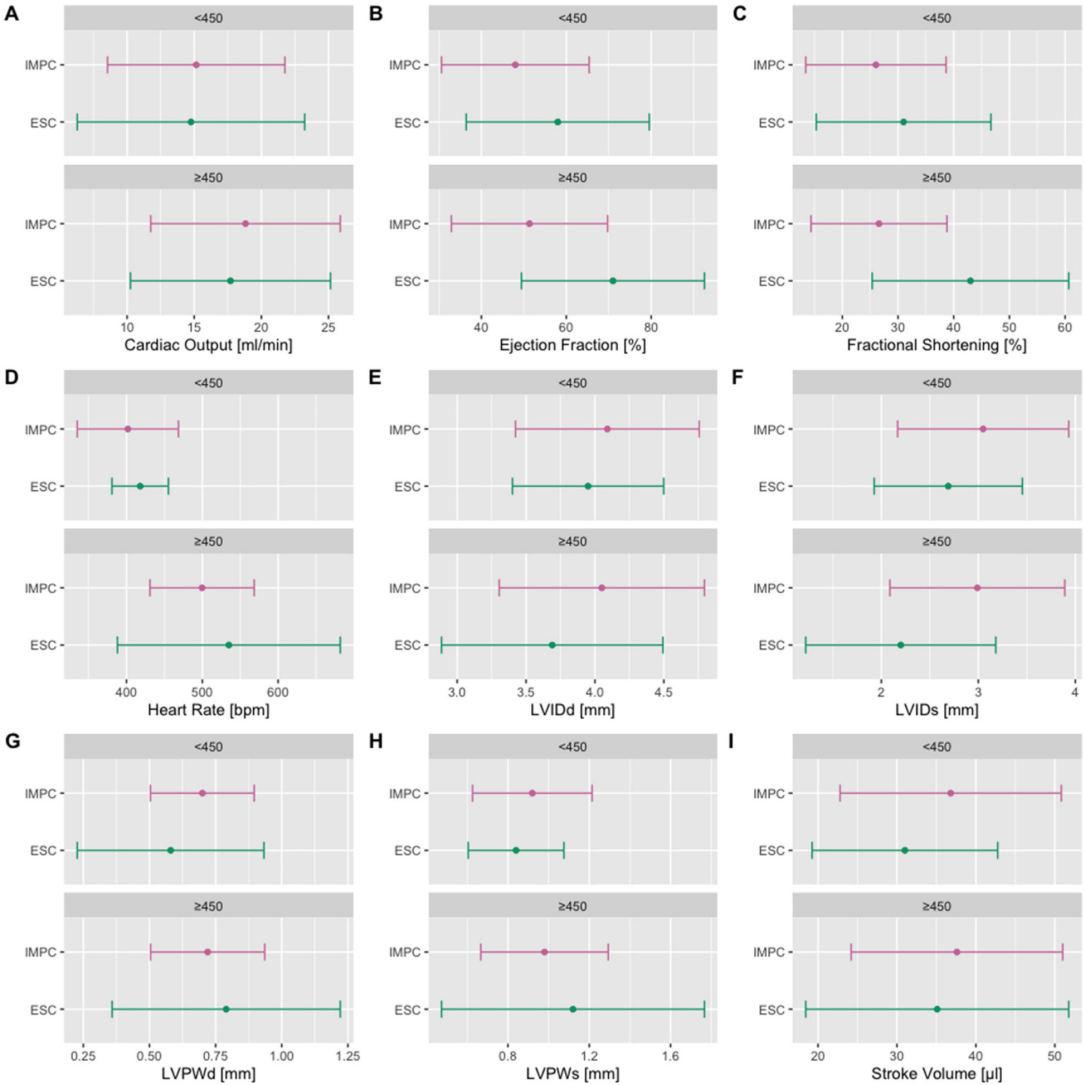
Independent, non-IMPC reference ranges of the ESC multicenter study including a total of 300 TTE examinations, stratified by HR <450 or >450 bpm, performed on control/healthy C57BL/6 adult mice across 10 different laboratories reported in “Towards standardization of echocardiography for the evaluation of left ventricular function in adult rodents: a position paper of the ESC Working Group on Myocardial Function” (9). IMPC mice were under isoflurane anesthesia, whereas ESC mice were under isoflurane or sevoflurane anesthesia. ESC mice were pooled for sex and ∼12 weeks of age, equivalent to the IMPC EA time point. In both sets, <450 or >450 bpm, CO, HR, LVPWd, LVPWs, and SV show a close alignment with the reference ranges generated herein. There are minor shifts that fall beyond the reference ranges generated herein for EF and FS and concurrently LVIDd and LVIDs. Color code: 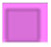 Reference ranges generated herein, and 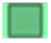 reference ranges of the ESC study, pooled for sex and stratified by <450 (upper panel) or >450 bpm (lower panel). LVIDd and LVIDs, left ventricular internal diameter in diastole and systole; LVPWd and LVPWs, posterior wall thicknesses in diastole and systole.

In each non-IMPC study, where suitable, we presented the data split by sex and overlaid with the sex-specific 95% reference range calculated herein for conscious (a collaborative cross study and non-IMPC studies at the GMC) or isoflurane-anesthetized mice (Jaxwest1 project, Eumorphia6 project, and ESC study). In the ESC study, we used mean ± 1.96 × SD to calculate 95% reference range, stratified by HR <450 or >450 bpm, and compared them with the 95% reference range calculated herein, equally stratified according to the method of the ESC study. Figure 8 shows the founder strain data from the collaborative cross study overlaid with the reference ranges split by sex, whereas Supplementary Figure S5 illustrates data from the Jaxwest1 project, Supplementary Figure S6 illustrates data from Eumorphia6, and Supplementary Figure S7 illustrates data from non-IMPC studies conducted at the GMC. Remarkably, and true for all TTE parameters, most non-C57BL/6N values lay within our reference ranges. There was a subset of outliers that fell outside of the reference ranges, which is to be expected with a heterogeneity of small size and phenotypic differences seen between inbred mouse strains. Figure 9 shows the ESC reference ranges overlaid with the reference ranges calculated herein, unclassified for sex. Remarkably, and true for all TTE parameters, most non-C57BL/6N values lay within our reference ranges. This visual representation of the reference ranges per value in the data was useful for revealing conformity to, and deviations from, the reference ranges calculated herein, for each parameter. In both sets, HR <450 or >450 bpm, there are minor shifts that fell beyond the reference ranges generated herein, which is to be expected with the heterogeneity of the ESC multicenter study of comparatively small mouse numbers and phenotypic differences between inbred mouse strains.

## Discussion

TTE is the imaging modality most widely used in cardiology (31). While TTE is non-invasive, it plays an important role in the diagnosis of numerous cardiovascular diseases, providing valuable qualitative and quantitative insights into prognosis and pathophysiological processes (32). The European Association of Cardiovascular Imaging and the American Heart Association embody an acknowledged expert consensus in cardiology, establishing regularly revised guidelines for the assessment and evaluation of echocardiography (31, 33). The availability of normal reference intervals for comparing individual patients is a cornerstone of echocardiography (34). Recent years have seen the publication of large, international, prospectively recruited studies with respect to age, gender, and ethnicity, expanding toward risk assessments (1, 5, 35–41).

Unlike other animals, mouse-specific reference ranges are uncommon (42–46), often highly specific to the mouse model (14) and typically derived from small sample sizes (15, 16).

In this multicenter study, we establish reference ranges using an exceptionally large TTE dataset comprising more than 15,000 wild-type control mice from the IMPC. Rather than being highly specific to a single mouse model, our approach incorporates C57/BL6N substrains, enabling broader applicability across multiple related substrains. The goal of the IMPC is to extend the functional annotation of the mammalian genome via the large-scale production and phenotypic characterization of single gene knockout mouse strains for all protein-coding genes. The phenotypic pipeline used to characterize these knockout strains includes cardiac morphological and functional assessment using TTE. For each knockout strain characterized, age, sex, and genetic background–matched wild-type control animals are also assessed. The TTE data from these C57BL/6N wild-type control mice hold extraordinary value and represent the focus of the current study.

Therefore, this study represents a large mouse dataset and allows the crucial understanding of the effects of sex, age, and anesthesia on echocardiograms in mice. To this end, we introduced a stepwise refinement of the data analysis and started with an in-depth assessment of the variability of 12 TTE parameters gathered from the IMPC. We identified nine clinically relevant TTE parameters that were highly robust and had low variability. Mainly for methodological reasons, the respiration rate with high variability was excluded here, but LVAWd and LVAWs were the exceptions. These parameters were collected only by a minority of centers and were therefore not included in the overall evaluation, but the values are made available in full in the Supplementary Materials.

The very nature of the multicenter, large-scale IMPC approach provides several confounding factors influencing the data. Understanding the impact of sex, age, body weight, and anesthesia on TTE is crucial for ensuring robust reference values. In this study, we were able to show by a relative importance analysis that anesthetics were the main predictor with the largest proportion of explained variance in the model. Consequently, the entire data must be split according to the anesthetic regime, as implemented here for conscious, anesthetized using isoflurane, or anesthetized using tribromoethanol states. Even with sex, body weight, and age contributing little to the variance, understanding the impact of these factors is crucial for ensuring robust reference ranges.

Evidence indicates that sex-related factors interact in generating differences in cardiovascular outcomes in humans (47) and mice (48). In the current study, male and female mice generally presented with comparable reference ranges with negligible sexual dimorphism. There may, however, be small sex differences for some parameters depending on the anesthetic agent. This observation is of key importance, and in part consistent with previous mouse data (48). While sexual dimorphism was not overtly apparent in inbred mice in the absence of any environmental, pharmacological, or genetic perturbations, the literature clearly supports sex differences in cardiovascular health, disease, and risk prevention (47, 49). Hence, our recommendation is that both sexes are included in any experimental design assuming that post-treatment, we may detect sex differences.

BW had only a minor influence on cardiac structure and function in healthy C57BL/6N mice. The weak correlations observed between BW and echocardiographic parameters indicate that BW-related changes primarily reflect physiological scaling with growth and aging rather than functional remodeling. Slight positive trends between BW and left ventricular dimensions suggested a mild, proportional enlargement of the heart, while FS and EF remained unchanged, confirming preserved systolic performance. No meaningful associations were found for wall thickness or stroke volume, reinforcing that BW is not a major determinant of cardiac function under healthy conditions. These results are consistent with the use of normal-weight mice maintained on a standard chow diet and provide a baseline reference framework for interpreting future studies in which body weight becomes a pathophysiological variable—such as in obesity, metabolic syndrome, or diabetes—where excess weight and metabolic dysregulation are known to substantially alter cardiac morphology and performance (4).

Volatile anesthetics induce a dose-dependent decrease in myocardial contractility and cardiac loading conditions in mice and humans (50, 51). These anesthetic effects reduce the metabolic activity and oxygen consumption of the heart. These protective properties can have a beneficial role to play in achieving myocardial oxygen balance during clinical anesthesia, surgery, and the postoperative phase (52–54). Our observation is that the presence of anesthesia matters. We confirm a decreased heart rate in anesthetized mice and go on to reveal distinctions in isoflurane inhalation anesthesia and intraperitoneal-injected tribromoethanol-induced anesthesia. These distinctions are of central importance and are impressively captured here. The known reduction in myocardial contractility under anesthesia is mirrored by increased left ventricular end-diastolic and end-systolic diameters and consequently decreased stroke volume, ejection fraction, and fractional shortening, independent of the age, anesthetic regime, and sex of the mice. Following the calculation of CO (CO = SV × HR), negligible anesthesia-related effects were anticipated, as LA mice exhibit a decrease in HR that is compensated by an increase in SV, resulting in comparable CO values to reference ranges. To emphasize the anesthesia distinctions, we map the effects of three different states (conscious, isoflurane, and tribromoethanol anesthesia) on nine TTE parameters in detail and present anesthesia-specific reference values.

Cardiac aging is a complex and naturally inescapable process denominated by various molecular hallmarks leading to left ventricular hypertrophy, fibrosis, and end-diastolic dysfunction (55), where the mouse closely recapitulates the human situation (56). Herein, we did observe a decrease in HR, an important determinant of sinoatrial node dysfunction with age (57), in 63-week-old mice (LA) compared with 12-week-old (EA) mice. Of interest, we identified age-related effects on end-systolic function and LV size. In particular, we observed enhancement in end-systolic and end-diastolic LV dimensions with preserved LV posterior wall thickness and a reduction in the LV capacity demonstrated by decreased ejection fraction and fractional shortening with increased stroke volume and cardiac output in conscious and isoflurane-anesthetized LA mice of both sexes. These age-related cardiac characteristics were, however, marginal, and rather attributable to disease-free cardiovascular aging with no signs of pathophysiological cardiac remodeling. An age-related decline in myocardial elasticity through collagen fiber remodeling, in combination with a drop in myocardial contractility caused by the diminution of myocardial cells and lower-efficiency calcium channels, provides rational explanations for the TTE parameter shifts observed herein, which is in agreement with previous mouse studies (58). To this end, we presented essential reference ranges that are critical for the assessment of normal age-related but disease-free cardiovascular ageing in mice (59, 60). Extending the aging period beyond 63 weeks, combined with interventions, could impact TTE parameters, although this was not part of the present study.

In the IMPC, we control for genetic diversity by using C57BL/6N inbred background substrains, thereby focusing our comparison on the genetic perturbation of interest, i.e., the single gene that is knocked out on this common genetic background. The transferability from the C57BL/6N background used here, however, was demonstrated by independently validating the ranges using data from a broad spectrum of non-IMPC C57BL/6N and C57BL/6J mice and other inbred and wild-derived inbred strains [(9, 20) https://phenome.jax.org/projects/Eumorphia6; https://phenome.jax.org/projects/Jaxwest1]. This validation indicates that C57BL/6N-based reference values represent a robust and comprehensive indicator of normality for many strains and can be used as a starting point for experimental investigations of cardiac function in the mouse. A subset of outlier strain-parameter combinations was identified; for example, LVIDs, LVIDd, EF, and FS in PWK/PhJ mice fell below the reported C57BL/6N-based reference range. The particularly small body weight of this wild-derived genetically diverse strain (20, 61) is consistent with a smaller but healthy heart and therefore explains the decreased LV inner dimensions.

This independent validation is also valid for reference values obtained in the ESC study using control/healthy C57BL/6 mice. A subset of outliers of the herein C57BL/6N-based reference values was identified; for example, LVIDs and LVIDd fell below the reference values and, as a consequence, EF and FS were above the ESC reference values. Underlying data differed in key points such as data volume (300 vs. 8,000 mice), anesthesia (isoflurane or sevoflurane vs. isoflurane anesthesia), and age range (8 weeks to 3 months vs. 12 weeks), potential factors that may explain the discrepancies between some parameter-specific reference values.

Each study comes with inherent limitations. In the present work, echocardiography was performed using 2D M-mode in the parasternal short-axis view only, as defined by the standardized IMPC phenotyping pipeline. Additional imaging modalities such as pulse-wave Doppler, tissue Doppler, speckle-tracking strain analysis, or three-dimensional echocardiography were not included in the high-throughput setting. The IMPC design prioritizes throughput, reproducibility, and comparability across international centers, which necessarily limits the inclusion of advanced modalities requiring operator-dependent optimization. Consequently, our analysis focused on traditional measures of left ventricular structure and systolic function (e.g., diameters, wall thickness, fractional shortening, and ejection fraction). While diastolic parameters could not be assessed, the consistency and scale of the dataset (>15,000 mice) provided a uniquely robust reference for these core indices. Future studies may extend this work by integrating Doppler- or strain-based functional measurements into complementary, high-resolution pipelines. End-diastolic and end-systolic anterior wall thicknesses (LVAWd and LVAWs) were not entirely recorded at all contributing centers, yet the data are provided in full in Supplementary Table S3, stratified for sex and age. Body temperature was measured in all anesthetized mice using a rectal probe and maintained within its physiological range (36–38 °C) using a dedicated heating pad. Supplementary Figure S1 presents partial body temperature data to showcase the overall echocardiography conditions under anesthesia.

Under tribromoethanol anesthesia, only a subset of TTE parameters (FS, HR, LVIDd, LVIDs, and LVPWd) could be included in the analysis. This restriction reflects variability in data acquisition protocols and completeness across IMPC centers, as tribromoethanol was used in a smaller number of sites and primarily during earlier phases of data collection.

Although the dataset for this anesthetic condition is smaller and limited to core parameters, it still comprises 450 mice (*n* = 227 female; *n* = 223 male), exceeding the recommended minimum sample size for reliable reference range estimation (62) and thus remains representative within the IMPC framework. Importantly, despite its smaller scope, this dataset provides additional evidence that the type of anesthesia markedly influences echocardiographic readouts, thereby reinforcing one of the key findings of this study. These observations highlight the necessity of careful anesthetic selection and standardization in experimental echocardiography and aim to raise awareness of anesthesia as a critical variable influencing cardiac functional assessment in preclinical research.

The reference ranges are limited to the techniques and anesthetics described and are not intended for other imaging methodologies, such as MRI, or other anesthetic agents, such as ketamine.

A lack of transferability from mouse models to patients often arises from the absence of standardized procedures and reference frameworks to ensure reliable and accurate assessment of cardiac function in mice. The reference ranges reported herein address this gap by providing quantitative, anesthesia-specific benchmarks for cardiac morphology and function based on more than 15,000 healthy, normal-weight C57BL/6N mice. These values can be used to demarcate typical ranges for experimental control groups of a given sex and age and thus serve as a quality assurance tool for preclinical echocardiographic studies.

While this study intentionally focused on healthy, non-intervention mice to establish a robust disease-free physiological baseline, the resulting standardized framework provides the essential foundation for interpreting functional alterations in disease or intervention models. Consequently, these reference ranges offer a critical resource for assessing echocardiographic changes resulting from pharmacological, environmental, or genetic perturbations within a consistent and comparable context.

## Summary

In contrast to previous review articles that qualitatively summarized the influence of anesthesia, sex, and age on murine cardiac function, the present study delivers a quantitative, multicenter reference framework derived from more than 15,000 wild-type C57BL/6N mice. These data define a disease-free physiological baseline from healthy, normal-weight animals on a standard chow diet and establish systematic, anesthesia-specific reference ranges stratified by sex, age, and body weight. Our analyses identify anesthesia as the dominant source of variability in echocardiographic parameters, while sex- and age-related effects are comparatively minor but measurable. Importantly, validation across independent datasets and related substrains confirms the robustness and transferability of these reference values, providing the research community with a standardized foundation for reliable and comparable cardiac phenotyping in preclinical models.

## Data availability statement

The datasets presented in this study can be found in online repositories. The names of the repository/repositories and accession number(s) can be found below: IMPC data base (https://www.mousephenotype.org/data), links to data:https://ftp.ebi.ac.uk/pub/databases/impc/, https://ftp.ebi.ac.uk/pub/databases/impc/all-data-releases/release-21.0/.

## Ethics statement

The animal study was approved by the following institutions: (1) Baylor College of Medicine (BCM) (Institutional Animal Care and Use Committee approved license AN-5896); (2) German Mouse Clinic, Helmholtz Zentrum München (GMC) (#144-10, 15-168); (3) Medical Research Council (MRC)—Harwell (HAR) (Animal Welfare and Ethical Review Body approved licenses 70/8015 and 30/3384); (4) Institute Clinique de la Souris, Mouse Clinical Institute (ICS) (#4789-2016040511578546v2); (5) Czech Centre for Phenogenomics (CCP) (AV CR 62/2016, Academy of Sci., Czech Rep.); and (6) MARC Nanjing University (#NRCMM9). The study was conducted in accordance with the local legislation and institutional requirements.
