# Correction: Establishing standardized transthoracic echocardiography reference ranges for mouse models: insights into the impact of anesthesia, sex, and age

**DOI:** 10.3389/fcvm.2026.1792916

**Published:** 2026-02-23

**Authors:** Manuela A. Oestereicher, Christopher S. Ward, Elida Schneltzer, Susan Marschall, Helmut Fuchs, Valerie Gailus-Durner, Ghina Bou About, Mohammed Selloum, Hamid Meziane, Michelle Stewart, Lydia Teboul, Clare Norris, Dale Pimm, Marina Kan, Federico López Gómez, Robert Wilson, Mayra Monroy, Sheraz Pasha, Eva Zabrodska, Jan Prochazka, David Pajuelo Reguera, Zuzana Nichtova, Yann Herault, Sara Wells, Helen Parkinson, Jason D. Heaney, Radislav Sedlacek, Xiang Gao, Martin Hrabe de Angelis, Nadine Spielmann

**Affiliations:** 1 Institute of Experimental Genetics and German Mouse Clinic, Helmholtz Center Munich, German Research Center for Environmental Health, Neuherberg, Germany; 2 Department of Integrative Physiology, Baylor College of Medicine, Houston, TX, United States; 3 Université de Strasbourg, CNRS, INSERM, Institute de la Clinique de la Souris, CELPHEDIA, PHENOMIN, Illkirch, France; 4 The Mary Lyon Centre and MRC Harwell, Harwell Campus, Oxfordshire, United Kingdom; 5 European Molecular Biology Laboratory-European Bioinformatics Institute, Wellcome Trust Genome Campus, Hinxton, Cambridgeshire, United Kingdom; 6 Advanced Technology Cores, Baylor College of Medicine, Houston, TX, United States; 7 Czech Centre for Phenogenomics, Institute of Molecular Genetics of the Czech Academy of Sciences, Prague, Czechia; 8 Department of Molecular and Human Genetics, Baylor College of Medicine, Houston, TX, United States; 9 SKL of Pharmaceutical Biotechnology and Model Animal Research Center, Collaborative Innovation Center for Genetics and Development, Nanjing Biomedical Research Institute, Nanjing University, Nanjing, China; 10 Chair of Experimental Genetics, School of Life Science Weihenstephan, Technische Universität München, Freising, Germany; 11 German Center for Diabetes Research (DZD), Neuherberg, Germany

**Keywords:** anesthesia effects on cardiac function, c57BL/6N mouse model, cardiovascular phenotyping, mouse cardiac reference ranges, transthoracic echocardiography

Affiliations 2–8 were in the wrong order. Affiliations 2–8 have been reordered.

The original version of this article has been updated.

